# Accuracy of non-invasive methods for assessing the progress of labor in the first stage: a systematic review and meta-analysis

**DOI:** 10.1186/s12884-022-04938-y

**Published:** 2022-08-01

**Authors:** Wan-Lin Pan, Li-Li Chen, Meei-Ling Gau

**Affiliations:** 1 School of Nursing, National Taipei University of Nursing and Health Sciences, Taipei, Taiwan; 2 Department of Nurse-Midwifery and Women Health, National Taipei University of Nursing and Health Sciences, No. 365, Ming-Te Road, Peitou, Taipei 11219 Taiwan

**Keywords:** Vaginal examination, Purple line, Intrapartum ultrasound, Systematic review, Meta-analysis, Sensitivity, Specificity

## Abstract

**Background:**

The World Health Organization in recent years has emphasized reducing the possibility of unnecessary interventions in natural childbirth, but little is known about the accuracy of non-invasive methods when assessing the progress of labor. This paper presents a literature review to assess strategies that support non-invasive methods for labor during the first stage. It evaluates the available evidence to provide the most suitable assessments and predictions that objectively identify the progress of low-risk labor during the first stage of labor.

**Methods:**

A search for relevant literature was conducted using the electronic databases of PubMed, CINAHL, Web of Sciences, the Cochrane Library, Scopus, Medline (OVID), and CEPS, with publications up to November 2021. Records were screened against pre-specified inclusion/exclusion criteria and the potential papers from Google Scholar were examined to identify additional papers that may have been missed. The Quality Assessment of Diagnostic Accuracy Studies-2 (QUADAS-2) tool was used to appraise the methodological quality of the included studies. The certainty of the evidence was assessed using the Grading of Recommendations Assessment, Development, and Evaluation (GRADE) approach. Two independent investigators extracted the review’s characteristics, and discrepancies were resolved by consensus. This review calculated individual and pooled sensitivity, specificity, and positive predictive values, which were exported to STATA (version 14; Stata Corp., College Station, TX) to represent the performance of diagnostic testing.

**Results:**

Our search returned 2283 reports of which 13 fulfilled the inclusion criteria, accounting for 2594 women. The subjects were divided into groups according to the diagnostic tests used to assess the progress of their labor, including appearance assessment and sonographic imaging parameters (head perineum distance, HPD; angle of progression, AOP, and other parameters). HPD pooled sensitivity was 0.74 (0.65–0.82), and specificity was 0.77 (0.69–0.84). The pooled diagnostic odds ratio (DOR) was 8.21 (4.67–14.41) and 10.34 (5.02–21.27), respectively. The results of subgroup analysis showed that the summary sensitivity and specificity were of medium accuracy overall. The quality of evidence as assessed with GRADE was low.

**Conclusion:**

Vaginal examination is an intrinsic element in the use of the partogram, while transperineal ultrasound can also be used as an auxiliary tool. However, the presence of publication bias within the parameters of ultrasound indicates that the diagnostic performance may be overestimated. Thus, randomized controlled trials or large-scale prospective cohort studies are necessary.

**Supplementary Information:**

The online version contains supplementary material available at 10.1186/s12884-022-04938-y.

## Background

Assessing labor progress is performed during intrapartum care to monitor that labor and childbirth progresses as expected and to identify deviations from the norm as early as possible, so as to intervene to minimize or avoid maternal or fetal problems [1]. However, there is a constant debate about how to guide healthcare providers to monitor a woman’s physiological changes and aid the decision-making and planning of care during childbirth [2]. The World Health Organization (WHO) has made a strong recommendation in favor of using a modified partograph and a recommendation of vaginal examination (VE) every four hours during the first stage of labor [3]. VE is an invasive procedure that is frequently used to evaluate the labor progress [4], but studies have found that the actual number of examinations is significantly more than recommended. VE often increases women's pain, ache, embarrassment, fear [5], infection [6] and chorioamnionitis [7].

In clinical practice, VE is a standard method to evaluate labor progression. However, there are also several methods for assessing the progress of labor, including the frequency and quality of uterine contractions, fetal descent by abdominal palpation, or sonographic assessment [8, 9]. Changes are observed in the ‘purple line’ [a red/purple discoloration that appears from the edge of the anus and extends to the top of the buttocks as labor progresses] [10] and/or the transverse diagonal of the Michaelis sacral area (it is believed that this area of bone moves backward during advanced labor, pushing out the wings of the ilea and increasing the pelvic diameter) [11] as well as the maternal behavioral cues and appearance [12] in low-risk women during labor. In recent years, scholars have proposed using ultrasound as the new gold standard for labor progress assessment, so that sonographic imaging can be achieved during labor to determine station and head position. When using ultrasound, the angle of progression (AOP), fetal head direction (HD), head perineum distance (HPD), progression distance (PD), head symphysis distance (HSD) [13], and occiput-spine angle (OSA) [14] can provide useful values and prediction models on the labor progress [15].

Despite the various techniques described above for assessing labor progress, the evidence for these methods remains unclear. Healthcare providers are challenged by the lack of less-invasive procedures to establish a correct assessment of the labor progress. Thus, this systematic review aims to evaluate and compare the available evidence on the accuracy of different methods, in order to help doctors, midwives, and other healthcare providers choosing the best suitable assessments for the objective identification of the labor progress in women during the first stage of labor, especially the active phase.

## Methods

### Search strategies

This systematic review was conducted in accordance with the Preferred Reporting Items for Systematic Reviews and Meta-Analysis (PRISMA) and was registered in PROSPERO (ID, CRD42021291173). The search was done through electronic databases up until November 8, 2021. The search was conducted in seven databases, PubMed, CINAHL, Web of Sciences, the Cochrane Library, Scopus, Medline (OVID), and CEPS (Chinese Electronic Periodical Services), to identify eligible studies based on pre-determined criteria. The main search was conducted using a search string (Additional file 1: shows the complete data of the search string).

The search strategy used the Boolean terms OR/AND and truncation, as follows: (labor women OR delivery women OR childbirth women OR intrapartum women) AND (non-invasive assess* OR routine vaginal exam* OR purple line OR behavior obs* OR verbal express* OR uterine contraction OR electrohysterography OR electrohysterogram OR uterine electromyography OR uterine monitoring OR external tocodynamometer OR transperineal ultraso* OR transperineal sonog* OR transabdominal ultraso* OR transabdominal sonog*) AND (labor progress* OR cervical dilatation OR fetal descent* OR head descent*). The reference list and Google scholar were also searched to identify any additional relevant studies.

### Eligibility criteria

This review used the study characteristics as the criteria, including participants, settings, index testing, and reference standards. The participants were low-risk women aged 18 or older during the first stage of labor. The settings included hospital delivery rooms. Index testing refers to the purple line, behavior observations, uterine contractions, verbal expressions, electrohysterography, and intrapartum ultrasound, covering the parameters of sensitivity, specificity, and positive predictive value. Detection evaluation was conducted on the labor progress, cervical dilatation, and fetal descent. The reference standard was vaginal examinations. For this review, the exclusion criteria are: doctor or midwife experience, an abnormal first stage of labor, tool development, and psychometric testing.

### Data extraction and synthesis

**Fig. 1:**
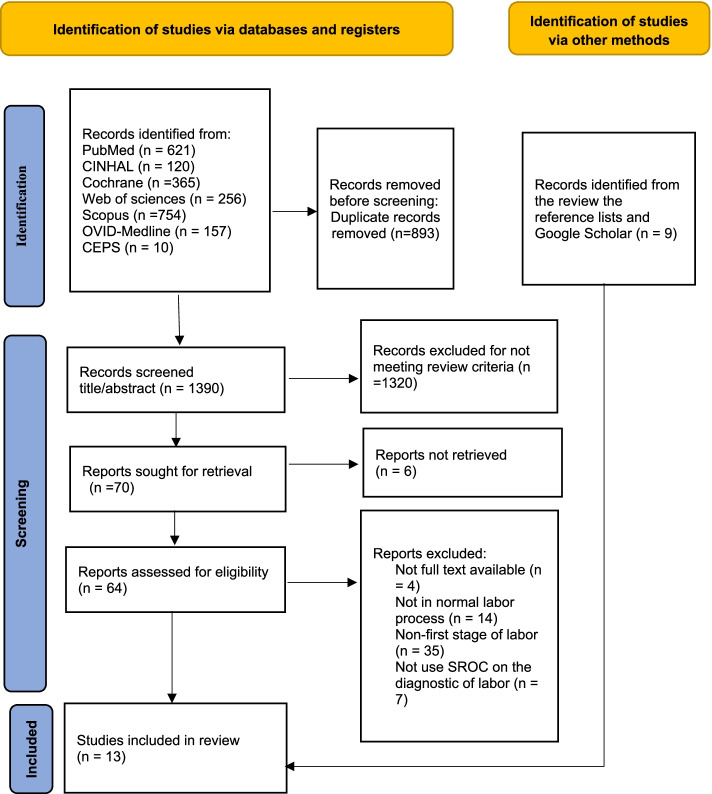
PRISMA 2020 Flowchart

The titles and abstracts of the studies were initially screened for eligibility. After the removal of duplicates, the titles and abstracts were screened by one reviewer [Pan]. Relevant full-text studies were then obtained and screened. Manuscripts that were potentially eligible for inclusion were discussed, and the two authors (Pan and Gau) must both agree on their inclusion or exclusion. The search was limited to studies published in peer-reviewed journals. A full flowchart of the study selection process using the PRISMA 2020 flowchart is illustrated in Fig. 1. When relevant data were not provided in an article, attempts were made to contact the corresponding author for clarification.

### Quality of evidence and risk of bias

There is currently no widely accepted checklist to assess the quality of non-invasive methods of assessment in labor progression. The Quality Assessment of Diagnostic Accuracy Studies 2 (QUADAS-2) tool to assess the quality of included studies. QUADAS-2 includes four key domains: patient selection, index tests, reference standard, and flow and timing. In addition, the first three items were also rated according to their applicability to the research question. The domains were rated as high, low, or unclear risk [16]. The Grading of Recommendations Assessment, Development and Evaluation (GRADE) methodology for diagnostic accuracy studies was used to assesses the quality of evidence of our pooled analyses across five domains, namely risk of bias, indirectness, inconsistency, imprecision, and publication bias [17]. These assessments were independently performed by two review authors (Pan and Gau), and any disagreements were resolved through consultation with a third reviewer.

### Data synthesis

The meta-analysis was performed by calculating pooled estimates of sensitivity, specificity, positive predictive value, true positives, true negatives, false positives, and false negatives, which can be extracted from all included studies. They were either reported directly or calculated from published studies. Heterogeneity was determined using both Cochran’s Q test and inconsistency index (*I*^2^) [18]. Here, *I*^2^ (which ranges from 0 to 100%) was used to examine the heterogeneity of results and to determine the analytical model [fixed-effects model or random-effects model].

An *I*^2^ value of > 50% and Cochran's Q test with a *p* value < 0.05 were defined as inter-study heterogeneity and were assessed by a random-effects model [19]. Next, the hierarchical model was adjusted to obtain a hierarchical summary receiver operator characteristic (HSROC) [20] curve with a 95% confidence interval [CI]. The model was constructed by plotting individual and summary points for diagnostic odds ratios (DOR) to assess overall diagnostic accuracy. Publication bias was assessed by Deek's funnel plot. STATA (version 14; Stata Corp., College Station, TX) was applied to represent the performance of diagnostic testing.

## Results

### Characteristics of the studies

Among all 2283 articles identified with the first search strategy, 893 overlapped (i.e., the same articles were identified using different search terms), and these records were exported to EndNote X8. Seven additional articles were identified by reviewing the reference lists of the existing relevant references. After screening the titles and abstracts, a total of 1390 studies was excluded. Except for 6 reports that were not retrieved, the text of 70 studies was retrieved for review. A further 60 studies were excluded because the full text was not available (*n* = 4), did not cover the normal labor process (*n* = 14), was not an assessment of the first stage of labor (*n* = 42), or did not use sensitivity and specificity on the diagnostic of labor (*n* = 7), all of which would have compromised the quality assessment process. The reference list search and Google scholar strategies identified 9 additional studies.

Three of the papers by Kordi et al. reported one study [10, 11, 21], and two of the papers by Wiafe et al. reported one study [22, 23]. In total, 13 quantitative studies were finally identified. Eight studies were conducted in Africa, three studies were conducted in Europe, one study was conducted in the Middle East, and one was conducted in Asia. Nearly 100% of the studies had a prospective observational design. One study had fewer than 50 participants, five studies had between 51 and 100 participants, and eight studies had more than 101 participants (Table 1).

**Table 1:** Publication, participants and aims of study

	Author, [year]	Year published		Country	Number of female	Method used to identify	Start measuring time	Main findings
1	Eid Farrag and Abd ElHamed Eltohamy [24]	2021		Egypt	120	Purple line	First stage of labor	The purple line appearance in the expectation of normal labor progress had 87.91% sensitivity, 39.53% specificity, and 85.25% accuracy
2	Kordi, Irani [11] [3 paper]	2013		Iran	350	Purple line and transverse diagonal of the Michaelis sacral	First stage of labor	The purple line and the transverse diagonal of the Michaelis sacral area can both be used to observe the labor progress, but the transverse diagonal of the Michaelis sacral area is a better predictor for observing the labor progress in comparison with the purple line
3	Elkadi, Ewida [25]	2021		Egypt	56	2D transperineal ultrasounds	Active phase	The angle of progression with cutoff value ≥ 97.0° had the highest predictive and diagnostic value, followed by the station of fetal head ≥ 0.0 followed by cervical dilatation ≥ 5 cm and cervical effacement ≥ 75%
4	Hjartardóttir, Lund [26]	2021		Ice land	99 nulliparous	3D transabdominal and transperineal ultrasound	Active phase	HPD and AOP are associated with spontaneous vaginal birth, and the ROC curves of 0.68 and 0.67 [best cutoff levels of ≥ 45 mm and ≤ 93 degrees]
5	Ibrahim, Nasr [27]	2021		Egypt	600 Primiparous [300 normal progress and 300 withprolonged 1st stage of labor	2D transperineal ultrasounds	Active phase	VE, HPD, and AOP are significantly correlated with one another and with both progress of labor and mode of birth [*p* value < 0.001]. When combining both HPD and AOP for the prediction of the mode of birth, the assessment of both parameters has a high sensitivity of 97.7% and a high positive predictive value of 86.63%
6	Mukdee, Suntharasaj [28]	2021		Thailand	330	2D transabdominal ultrasound	Active phase	The sensitivity of an OSA ≥ 100 degrees for predicting vaginal birth is 83.7%, but its specificity is only 17.1%. The combination of an OSA ≥ 100 degrees with multiparity and no induction of labor can predict vaginal birth with a positive likelihood ratio of 3.6
7	Fahmy, Elhalaby [29]	2020		Egypt	70 nulliparous	2D Transperineal ultrasound	Active phase	VE, HPD [≥ 120 degrees] and AOP [≤ 45 mm] are significantly correlated with one another and prediction of the occurrence of normal vaginal birth
8	Kandil, Elhalaby [30]	2020		Egypt	80	2D Transperineal ultrasound	Active phase	The sensitivity of an AOP > 104 degrees for predicting vaginal delivery is 90%, and the specificity is 86%
9	Solaiman, Atwa [31]	2020		Egypt	28	2D Transperineal ultrasound	Prolonged active phase of first or second stage s of labor	Using a cut off value of 115 degrees for the AOP, the positive predictive value [PPV] of vaginal birth is 87%; using a cut off value of 42 mm for HPD results in a PPV for vaginal birth of 85%
10	Maged, Soliman [14]	2019		Egypt	400	2D Transabdominal ultrasound	Active phase	An OSA < 126 degrees has a sensitivity, specificity, and accuracy of 82%, 64.6%, and 92% in the prediction of the mode of birth, respectively
11	Wiafe, Whitehead [23]	2018		Ghana	201	2D Transperineal Ultrasound	Active phase	An HPD of 3.6 cm is the cut-off value for the high likelihood of predicting an engaged fetal head. An AOP of 101 degrees is consistent with engagement by VE
12	Eggebø, Hassan [32]	2014	UK and Norway		150	2D and 3D transabdominal and Transperineal ultrasound	Prolonged first stage of labor	The area under the ROC curve for the prediction of vaginal birth is 81% using HPD as the test variable and 72% using AOP. Multivariable logistic regression analysis showed that an HPD ≤ 40 mm, an AOP ≥ 110 degrees, a non-occiput posterior position, and the spontaneous onset of labor are independent predictors for vaginal birth
13	Torkildsen, Salvesen [33]	2011	Norway		110 primiparous	2D and 3D Transperineal Ultrasound	prolonged first stage of labor	For women in prolonged labor, HPD and AOP can predict vaginal birth with the ROC curve of 0.81 and 0.76. The 2D and 3D acquisitions were similar

*2D* two-dimensional ultrasound, *3D* three-dimensional ultrasound, *OSA* Occiput-spine angle, *HPD* Head-perineum distance, *AOP* Angle of progression, *HD* Fetal head direction, *ROC* Curve, receiver operating characteristic curve

### Quality of the studies

**Fig. 2:**
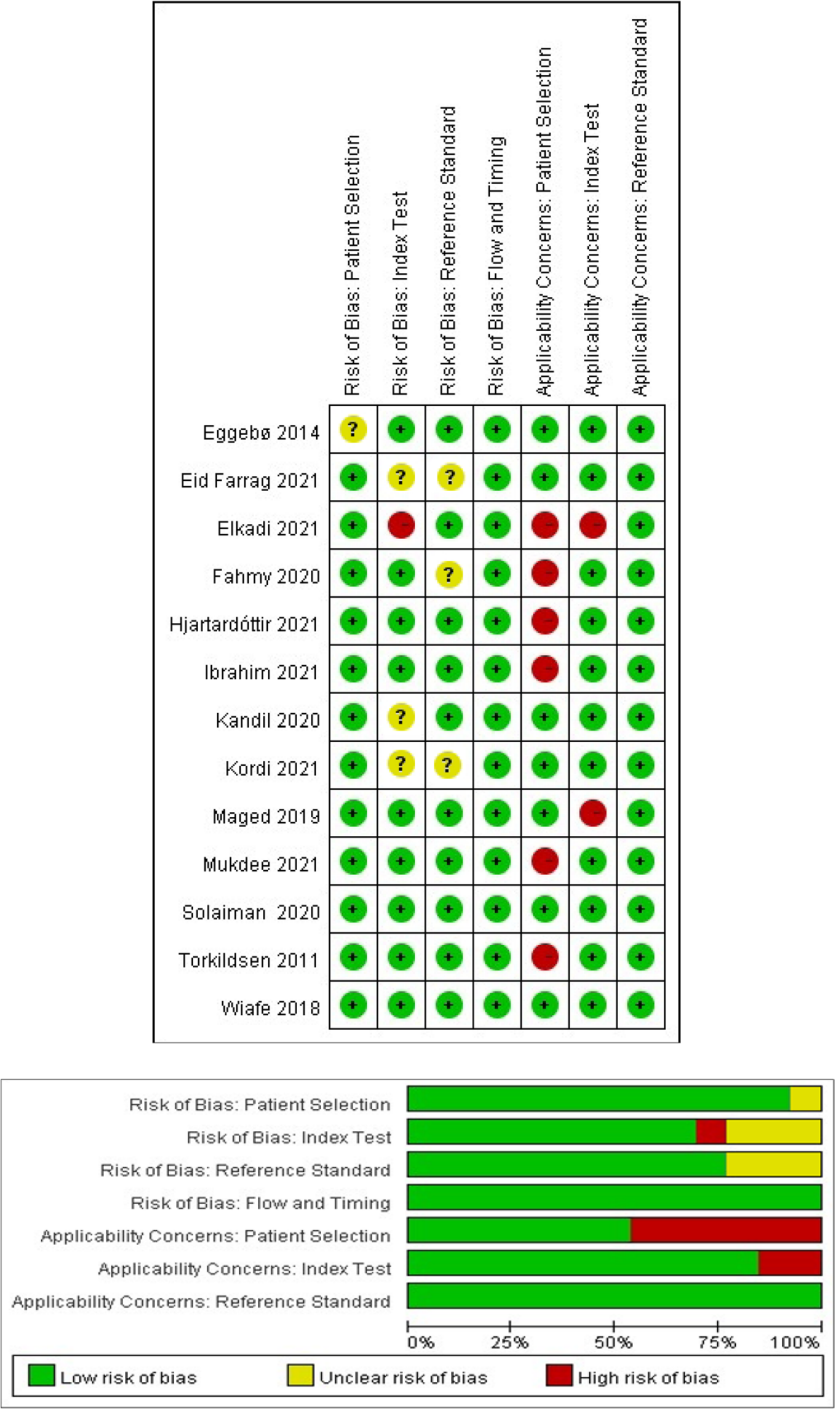
QUADAS-2 quality assessment of included studies

The QUADAS-2 quality assessment of the included studies is shown in Fig. 2. Among the 13 studies, one study indicated unclear patient selection bias, because the exclusion criteria were not specified; four studies reported that the index test was sufficiently detailed or not performed before the reference index; and three studies did not illustrate VE or labor progression, thus indicating that the reference test standard bias is unclear. Flow and timing projects reported well. Regarding the applicability of the study to the review question, five articles focused on primiparas only, and one excluded occipital posterior position. Due to the above reasons, these studies were assessed as being high risk of patient selection. No study raised concerns about the reference standard or patient selection (Fig. 2). Because the non-invasive method standard has been published to date to assess labor progress in the first stage, no threshold value of the included 13 studies was prespecified. The quality of all the included studies was moderate, and all satisfied at least 7 of the 12 items.

### Systematic review of the overall assessment accuracy

**Fig. 3:**
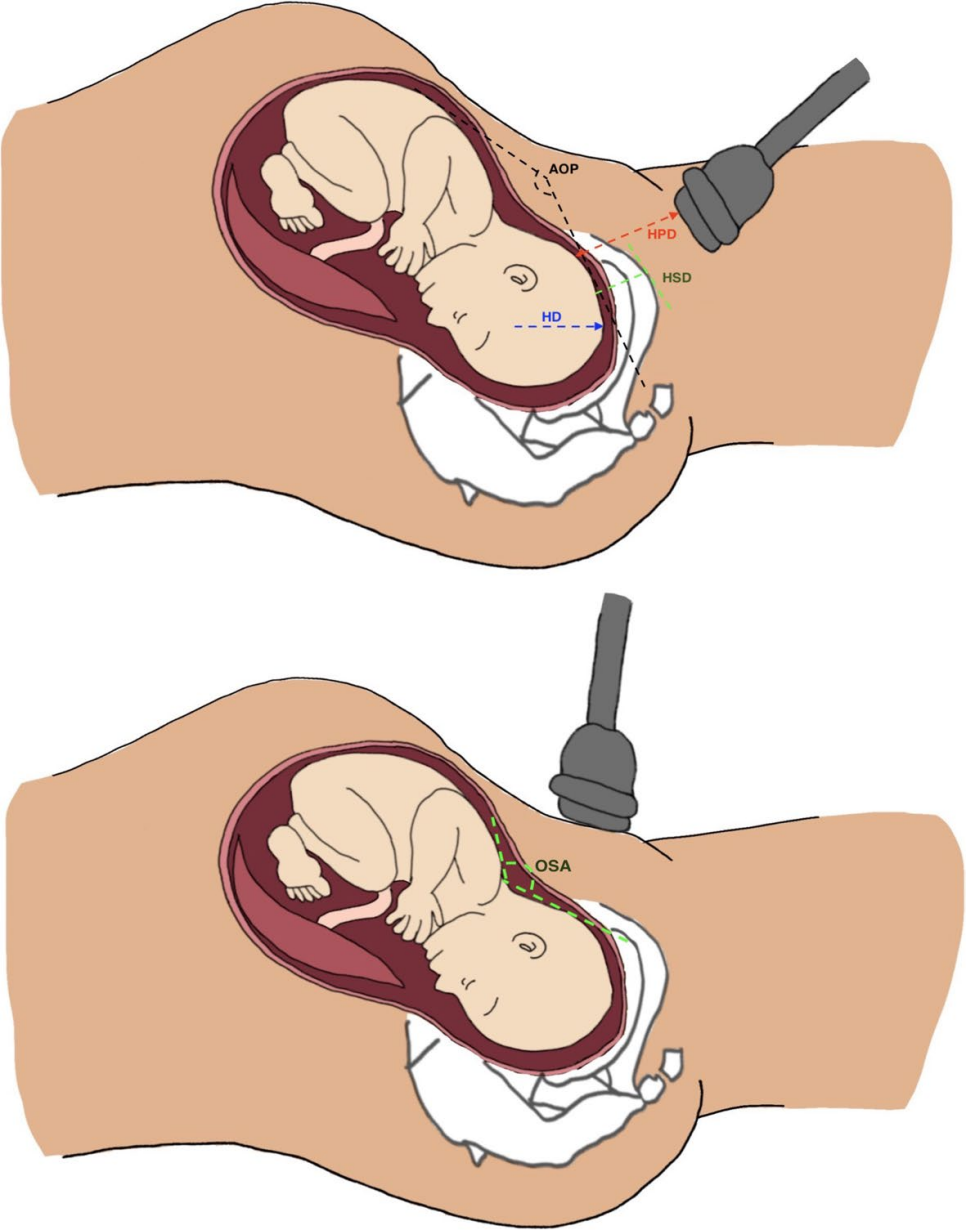
The OSA, HPD, AOP, HD and HSD of ultrasound parameters. OSA, occiput-spine angle; HPD, head-perineum distance; AOP, angle of progression; HD fetal head direction; HSD, head symphysis distance

The studies were divided into groups according to the diagnostic test used to assess the progress of labor. In total, two studies evaluated the accuracy of the appearance of the purple line for assessing labor progress, and eleven studies evaluated the use of 2D and/or 3D transabdominal and/or transperineal ultrasound, which OSA, HPD, AOP, HD, and HSD (Fig. 3) can provide useful values and prediction models on how labor will progress (Additional file 3_ sensitivity and specificity row data).

### Subgroup analysis for appearance assessment

The purple line appeared on women during the active phase of labor with 68.57–87.91% sensitivity and 42.66–39.53% specificity [11, 24], and the line was more obvious in women of lighter skin color than in women who have darker skin coloring. Kordi and Irani [11] added the transverse diagonal of the Michaelis sacral area as an evaluation item for labor, and also found its sensitivity was better than the purple line, which is probably because it is not affected judge by skin color.

### Subgroup analysis for the assessment accuracy of transabdominal and transperineal ultrasound

Intrapartum ultrasound can provide objective and quantitative labor data, including the occiput-spine angle, head-perineum distance, angle of progression, fetal head direction, and head symphysis distance.

### Occiput-spine angle

Transabdominal ultrasound can be used to detect the head deflexion OSA during the labor progress. OSA > 100 degrees up to 126 degrees can predict normal vaginal birth, while < 126 degrees indicate a significantly longer duration of both the first and second stages of labor and a higher rate of requiring a cesarean section [14, 28].

### Head-perineum distance

HPD can be used to predict an engaged fetal head, evaluate the progress of labor, and predict the mode of birth. When the cut-off point is 36–46 mm, the labor is more likely to proceed in a normal pattern, with 61–96% sensitivity and 63–91% specificity.

### Angle of progression

AOP data can be used to evaluate the progress of labor and predict the mode of birth When AOP is 93–120 degrees, labor is more likely to progress as normal, with 52–92% sensitivity and 46–86% specificity.

### Other parameters

HD and HSD are different intrapartum transperineal ultrasound parameters. Fahmy and Elhalaby [29] used HD (> 30 degrees) as the cut-off point to predict an engaged fetal head. Wiafe and Whitehead [23] used HSD (≤ 28 mm) as the predictive parameters of vaginal childbirth.

### Quantitative data synthesis of HPD and AOP

**Fig. 4:**
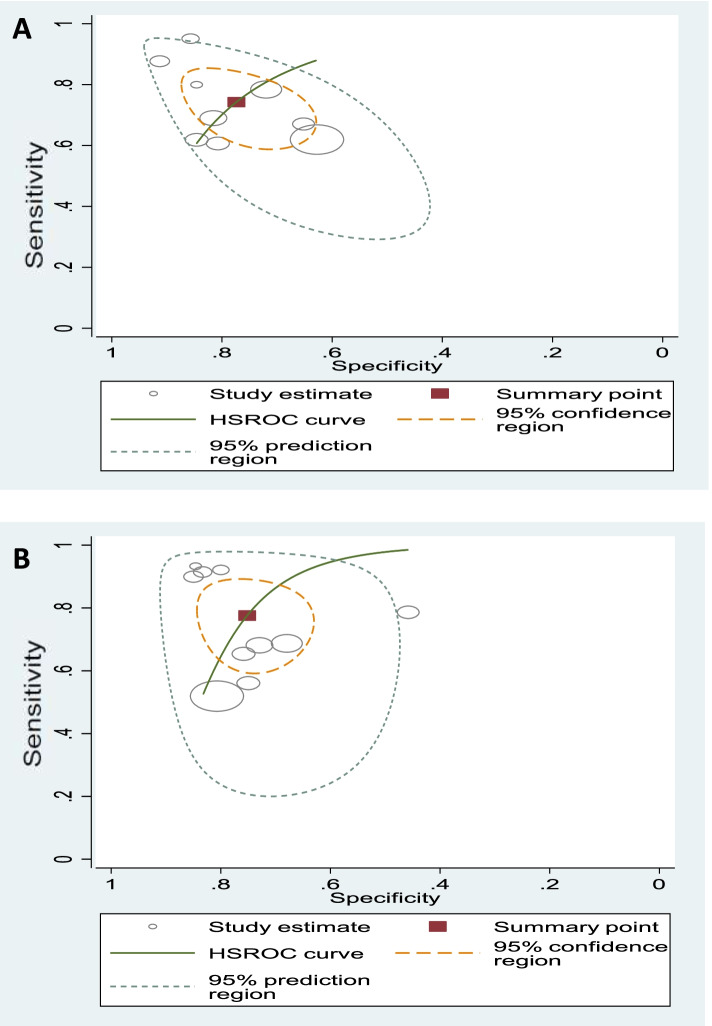
HSROC curve for the diagnostic accuracy of HPD and AOP

Since data on HPD and AOP parameters for data synthesis was available in three studies only, these two parameters only are discussed here. The pooled sensitivities and 95% confidence interval (CI) for HPD and AOP were 0.74 (0.65–0.82) and 0.78 (0.66–0.86), respectively, the specificities and 95% CIs were 0.77 (0.69–0.84) and 0.75 (0.67–0.82), respectively, while AUC for HPD and AOP were 0.83 and 0.81. The HSROC curve was obtained by using the hierarchical regression model to present an overall summary of HPD and AOP and lies to the left of the diagonal (Fig. 4). It indicates the fact that HPD and AOP have comparable accuracy for the diagnosis of labor progress.

### Investigation for heterogeneity

**Fig. 5:**
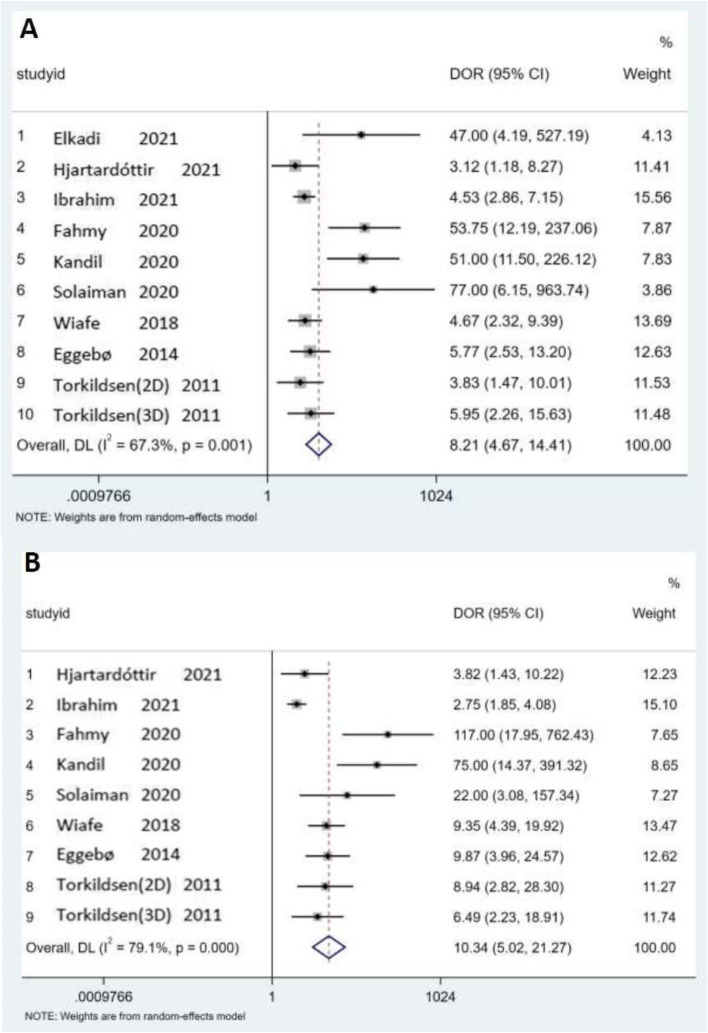
Forest plots for the diagnostic accuracy of HPD and AOP. DOR: diagnostic odds ratio; *I*2: percentage of total variation across studies due to heterogeneity rather than chance

Given the small number of included studies, the forest plots were just from HPD and AOP, in which the summary from nine data subsets of eight studies and ten data subsets of nine studies are shown in Figs. 5A and B, respectively. No statistically significant difference was observed between HPD and AOP when exploring the threshold effect, while the Spearman correlation coefficient was determined to be 0.46 (*p* = 0.21) and 0.23 (*p* = 0.53). Meta-analysis executed the pooling of odds ratios using the random-effects inverse-variance model. The pooled DOR of HPD and AOP were 8.21 (95% CI, 4.67–14.41) and 10.34 (95%, CI, 5.02–21.27), respectively. There was significant heterogeneity of AOP (Cochran's Q = 27.50; *p* < 0.001; *I*^2^ = 67.3%) and AOP (Cochran's Q = 38.32; *p* < 0.0001; *I*^2^ = 79.1%).

### Publication biases

**Fig. 6:**
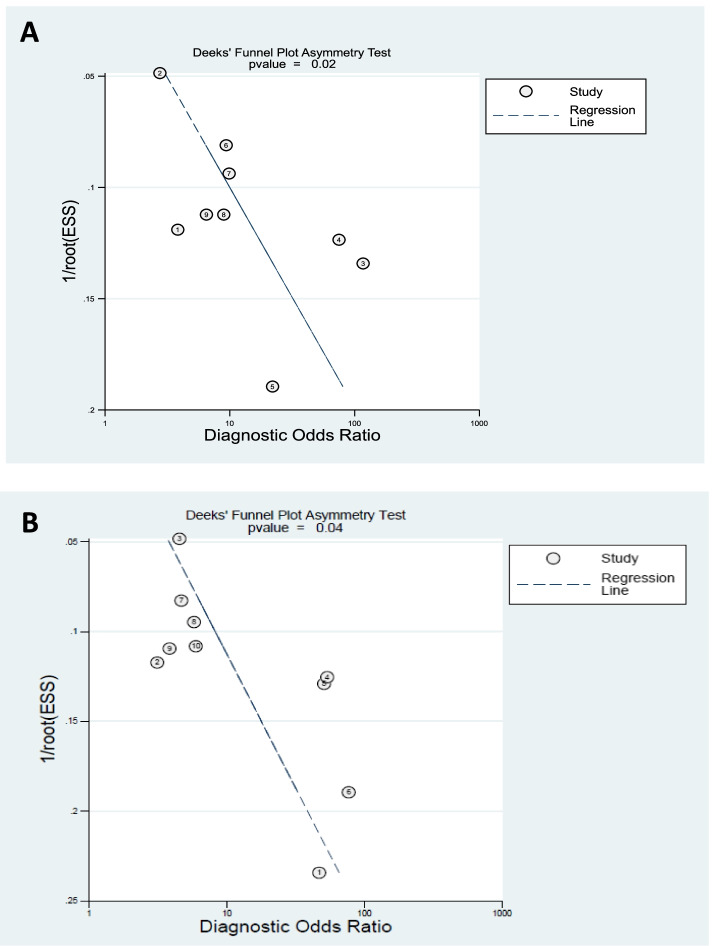
Deeks’ funnel plot of publication bias of HPD and AOP. ESS, effective sample size

We detected Deeks’ funnel plot of publication bias. Asymmetrically distributed studies with the regression line’s coefficient for HPD (*p* value = 0.02) and AOP (*p* value = 0.04) indicated publication bias was present (Fig. 6).

The overall quality of evidence presented in those studies was considered low to very low, according to the GRADE tool assessment. Two subgroups were very low on the appearance assessment and OSA parameters on ultrasound, and the other four parameters on ultrasound were low. The evidence was of quality due to study design limitations (cross-sectional design and case selection bias), indirectness, and inconsistency (small sample size and high heterogeneity) (Additional file 2_ GRADE tool assessment).

## Discussion

This systematic literature search revealed 13 studies that evaluate the diagnostic performance by the purple line, the transverse diagonal of the Michaelis sacral area and transabdominal and transperineal ultrasounds, as auxiliary tools to assess the first stage of labor progress. The results showed the ultrasound parameters, in which HPD and AOP were a medium sensitive 0.74 (0. 65–0.82) and 0.78 (0.66–0.86) and specificities 0.77 (0.69–0.84) and 0.75 (0.67–0.82) compared with VE as the current clinical gold standard. The pooled DOR was 8.21and 10.34, indicating that the HPD and AOP were classified as having good normal birth progress tool parameters. However, significant heterogeneity was found between the studies. The quality of evidence assessed with GRADE was low to very low.

For the purple line subgroup, the quality of the trial was very low according to GRADE, although accurately this should be, and only two studies reporting on the purple line studies were included. Although past research has found the purple line positively correlated with cervical dilatation and fetal head descent in labor [10, 21, 24, 34, 35], the specificity between VE and the purple line in labor was poor in laboring women. This could lead to a test result indicating healthy women having abnormal labor progress.

Eleven of the included 13 studies compared transabdominal and/or transperineal ultrasound, and several different ultrasound measurements have been proposed. Most studies used the AOP and HPD parameters. because they were easy to perform [36]. However, we found that pooled sensitivity and specificity were moderate, considerable high between-study heterogeneities were observed. This finding was partly attributable to the small sample size, different ultrasound systems, different ethnicity, or other study-specific covariates. Moreover, significant publication bias was present, and the diagnostic performance may have been limited by the significant heterogeneity between studies and the low quality of the GRADE.

More and more information about the use of ultrasound for the assessment of labor progress has been accumulated in recent years, and it has become a relatively new way of assessing childbirth [37]. Transperineal ultrasound parameters (HPD and AOP) significantly correlated with each other as well as with both labor progress and mode of birth [38–43]. Chan and Ng [41] suggested that a combination of AOP and HPD can increase their predictive potential of normal vaginal birth and sensitivity to 97.7%. However, ultrasound cannot replace the clinical assessment of cervical dilatation at late stages [22]. Subgroup analysis showed that in women with prolonged labor, the sensitivity and specificity of ultrasound appeared to be poor [32, 33]. This might suggest that an ultrasound is not a very good predictor for women with prolonged labor.

There are limitations to this study. The very low to low quality of the evidence is of major concern mainly due to selection bias as well as verification bias. First, the aim of the paper was to achieve a synthesis of the sensitivity, specificity, positive predictive value, and negative predictive value, which may limit the analyses of the different research methods adopted. Next, in this review, there are eight studies from the same African country, and because there are different healthcare institutions, ultrasound systems and observers in conducting births may have contributed to the variability of the results [36, 44]. Moreover, pelvic shapes and sizes differ with ethnicity, which may result in different results in the accuracy of labor assessment. Furthermore, there are several studies with small sample sizes, and therefore, selection bias may have been present.

The contribution of this systematic review is that clinical practice could consider including ultrasound as an adjunct to assessing labor progress during labor. In addition to being non-invasive, previous studies have found that evaluating labor progress through VE remains one of the most challenging skills for younger midwives or midwifery students [45]. This review found that the roles of HPD (93°-120°) and AOP (36–46 mm) in transperineal ultrasounds had sensitivity ranging from 0.74 to 0.78. In other words, it could potentially identify over 70% of normal labor progress. Some studies even mentioned the feasibility of using portable ultrasound [26]*.* Therefore, in clinical practice, HPD and AOP parameters can be combined with VE, which will help to estimate the progress of low-risk normal birth.

## Conclusion

The results of this meta-analysis support the international recommendations of WHO and confirmed that the HPD and AOP parameters are promising methods for use in transperineal ultrasound for decreased vaginal examination in the first stage of labor. Although these studies are not randomized controlled trial designs, they demonstrated that the benefits to women and healthcare givers are unequivocally clear. Due to publication bias in this study, future research is suggested to employ randomized controlled trials or large-scale prospective cohort studies, and focus on test combinations with VE in order to improve the predictive accuracy in normal labor progress.

## Supplementary Information

**Additional file 1.** Search strategy.

**Additional file 2.** Study Quality of Evidence According to Guidelines.

**Additional file 3.** Sensitivity and specificity row data.
