# Supplementary material for: Accuracy of non-invasive methods for assessing the progress of labor in the first stage: a systematic review and meta-analysis
Source: BMC Pregnancy Childbirth. 2022 Aug 1;22:608. doi: 10.1186/s12884-022-04938-y (PMC9341104; doi:10.1186/s12884-022-04938-y)
Supplement: Supplementary file 1 — Additional file 1. Search strategy. [file 12884_2022_4938_MOESM1_ESM.docx]

Appendix I: Search strategy

PubMed

| # | Query |  |
| --- | --- | --- |
| S1 | Labor and women.mp. | 124,819 |
| S2 | Delivery and women.mp. | 109,150 |
| S3 | Childbirth and women.mp. | 23,664 |
| S4 | Intrapartum and women.mp. | 4,990 |
| S5 | S1 or S2 or S3 or S4 | 213,023 |
| S6 | Non-invasive assessment.mp. | 30,531 |
| S7 | Routine vaginal exam.mp. | 386 |
| S8 | Purple line.mp. | 482 |
| S9 | Behavior observation.mp. | 22,437 |
| S10 | Uterine contraction.mp. | 10,020 |
| S11 | Verbal expression.mp. | 5,330 |
| S12 | Electro hysterography | 94 |
| S13 | Electrohysterogram | 115 |
| S14 | Uterine electromyography | 637 |
| S15 | Uterine monitoring | 4,771 |
| S16 | Uterine contraction | 10,020 |
| S17 | External tocodynamometer | 32 |
| S18 | Transperineal ultrasond | 2,029 |
| S19 | Transperineal sonography | 1,274 |
| S20 | Transabdominal ultrasond | 4,502 |
| S21 | Transabdominal sonography | 3,493 |
| S22 | S6 or S7 or S8 or S9 or S10 or S11 or S12 or S13 or S14 or 15 or S16 or S17 or S18 or S19 or S20 or S21 | 80,048 |
| S23 | Fetal descent.mp. | 769 |
| S24 | Cervical dilatation.mp. | 5,041 |
| S25 | Labor progress* | 73,006 |
| S26 | Head descent | 849 |
| S27 | S23 or S24 or S25 or S26 | 80,470 |
| S28 | S5 and S22 and S27 | 621 |

CINAHL

| # | Query |  |
| --- | --- | --- |
| S1 | Labor and women.mp. | 13,787 |
| S2 | Delivery and women.mp. | 28,679 |
| S3 | Childbirth and women.mp. | 13,540 |
| S4 | Intrapartum and women.mp. | 2,031 |
| S5 | S1 or S2 or S3 or S4 | 41,239 |
| S6 | Non-invasive assessment.mp. | 3,100 |
| S7 | Routine vaginal examination.mp. | 122 |
| S8 | Purple line.mp. | 47 |
| S9 | Behavior observation.mp. | 8,366 |
| S10 | Uterine contraction.mp. | 1,030 |
| S11 | Verbal expression.mp. | 898 |
| S12 | Electrohysterography | 13 |
| S13 | Electrohysterogram | 7 |
| S14 | Uterine electromyography | 55 |
| S15 | Uterine monitoring | 473 |
| S16 | Uterine contraction | 1,030 |
| S17 | External tocodynamometer | 8 |
| S18 | Transperineal ultrasound | 356 |
| S19 | Transperineal sonography | 37 |
| S20 | Transabdominal ultrasond | 488 |
| S21 | Transabdominal sonography | 86 |
| S22 | S6 or S7 or S8 or S9 or S10 or S11 or S12 or S13 or S14 or 15 or S16 or S17 or S18 or S19 or S20 or S21 | 14,724 |
| S23 | Fetal descent.mp. | 142 |
| S24 | Cervical dilatation.mp. | 837 |
| S25 | Labor progress | 784 |
| S26 | Head descent | 105 |
| S27 | S23 or S24 or S25 or S26 | 1,741 |
| S28 | S5 and S22 and S27 | 120 |

Web of Science (WOS)

| # | Query |  |
| --- | --- | --- |
| S1 | Labor and women.mp. | 47,802 |
| S2 | Delivery and women.mp. | 78,583 |
| S3 | Childbirth and women.mp. | 17,246 |
| S4 | Intrapartum and women.mp. | 4,427 |
| S5 | S1 or S2 or S3 or S4 | 129,374 |
| S6 | Non-invasive assessment.mp. | 16,127 |
| S7 | Routine vaginal exam.mp. | 532 |
| S8 | Purple line.mp. | 2,271 |
| S9 | Behavior observation.mp. | 162,431 |
| S10 | Uterine contraction.mp. | 4,993 |
| S11 | Verbal expression.mp. | 5,224 |
| S12 | Electrohysterography | 105 |
| S13 | Electrohysterogram | 101 |
| S14 | Uterine electromyography | 226 |
| S15 | Uterine monitoring | 3,456 |
| S16 | Uterine contraction | 4,994 |
| S17 | External tocodynamometer | 21 |
| S18 | Transperineal ultrasond | 1,915 |
| S19 | Transperineal sonography | 249 |
| S20 | Transabdominal ultrasond | 2,957 |
| S21 | Transabdominal sonography | 879 |
| S22 | S6 or S7 or S8 or S9 or S10 or S11 or S12 or S13 or S14 or 15 or S16 or S17 or S18 or S19 or S20 or S21 | 194,964 |
| S23 | Fetal descent.mp. | 695 |
| S24 | Cervical dilatation.mp. | 2,281 |
| S25 | Labor progress* | 6,298 |
| S26 | Head descent | 919 |
| S27 | S23 or S24 or S25 or S26 | 9,774 |
| S28 | S5 and S22 and S27 | 256 |

The Cochrane Library

| # | Query |  |
| --- | --- | --- |
| S1 | Labor and women.mp. | 8,929 |
| S2 | Delivery and women.mp. | 16,913 |
| S3 | Childbirth and women.mp. | 3,284 |
| S4 | Intrapartum and women.mp. | 885 |
| S5 | S1 or S2 or S3 or S4 | 21,217 |
| S6 | Non-invasive assessment.mp. | 5,863 |
| S7 | Routine vaginal exam.mp. | 298 |
| S8 | Purple line.mp. | 17 |
| S9 | Behavior observation.mp. | 18,920 |
| S10 | Uterine contraction.mp. | 1,709 |
| S11 | Verbal expression.mp. | 870 |
| S12 | Electrohysterography | 4 |
| S13 | Electrohysterogram | 0 |
| S14 | Uterine electromyography | 12 |
| S15 | Uterine monitoring | 1,031 |
| S16 | Uterine contraction | 1,709 |
| S17 | External tocodynamometer | 7 |
| S18 | Transperineal ultrasond | 0 |
| S19 | Transperineal sonography | 5 |
| S20 | Transabdominal ultrasond | 0 |
| S21 | Transabdominal sonography | 42 |
| S22 | S6 or S7 or S8 or S9 or S10 or S11 or S12 or S13 or S14 or 15 or S16 or S17 or S18 or S19 or S20 or S21 | 32,432 |
| S23 | Fetal descent.mp. | 48 |
| S24 | Cervical dilatation.mp. | 2104 |
| S25 | Labor progress* | 778 |
| S26 | Head descent | 31 |
| S27 | S23 or S24 or S25 or S26 | 2,728 |
| S28 | S5 and S22 and S27 | 365 |

scopus

| # | Query |  |
| --- | --- | --- |
| S1 | Labor and women.mp. | 85,557 |
| S2 | Delivery and women.mp. | 118,878 |
| S3 | Childbirth and women.mp. | 23,030 |
| S4 | Intrapartum and women.mp. | 5,359 |
| S5 | S1 or S2 or S3 or S4 | 181,030 |
| S6 | Non-invasive assessment.mp. | 33,949 |
| S7 | Routine vaginal exam.mp. | 77 |
| S8 | Purple line.mp. | 1,757 |
| S9 | Behavior observation.mp. | 216,567 |
| S10 | Uterine contraction.mp. | 15,450 |
| S11 | Verbal expression.mp. | 9,455 |
| S12 | Electrohysterography | 208 |
| S13 | Electrohysterogram | 210 |
| S14 | Uterine electromyography | 670 |
| S15 | Uterine monitoring | 8,453 |
| S16 | Uterine contraction | 15,450 |
| S17 | External tocodynamometer | 39 |
| S18 | Transperineal ultrasound | 1,850 |
| S19 | Transperineal sonography | 189 |
| S20 | Transabdominal ultrasond | 3,533 |
| S21 | Transabdominal sonography | 882 |
| S22 | S6 or S7 or S8 or S9 or S10 or S11 or S12 or S13 or S14 or 15 or S16 or S17 or S18 or S19 or S20 or S21 | 287,576 |
| S23 | Fetal descent.mp. | 827 |
| S24 | Cervical dilatation.mp. | 6,086 |
| S25 | Labor progress* | 9,598 |
| S26 | Head descent | 990 |
| S27 | S23 or S24 or S25 or S26 | 16,936 |
| S28 | S5 and S22 and S27 | 754 |

CEPS

| # | Query |  |
| --- | --- | --- |
| S1 | Labor and women.mp. | 3,463 |
| S2 | Delivery and women.mp. | 6,021 |
| S3 | Childbirth and women.mp. | 1,367 |
| S4 | Intrapartum and women.mp. | 196 |
| S5 | S1 or S2 or S3 or S4 | 4,858 |
| S6 | Non-invasive assessment.mp. | 483 |
| S7 | Routine vaginal exam.mp. | 134 |
| S8 | Purple line.mp. | 202 |
| S9 | Behavior observation.mp. | 4,660 |
| S10 | Uterine contraction.mp. | 890 |
| S11 | Verbal expression.mp. | 396 |
| S12 | Electrohysterography | 3 |
| S13 | Electrohysterogram | 5 |
| S14 | Uterine electromyography | 7 |
| S15 | Uterine monitoring | 371 |
| S16 | Uterine contraction | 890 |
| S17 | External tocodynamometer | 0 |
| S18 | Transperineal ultrasond | 311 |
| S19 | Transperineal sonography | 48 |
| S20 | Transabdominal ultrasond | 635 |
| S21 | Transabdominal sonography | 116 |
| S22 | S6 or S7 or S8 or S9 or S10 or S11 or S12 or S13 or S14 or 15 or S16 or S17 or S18 or S19 or S20 or S21 | 18877 |
| S23 | Fetal descent.mp. | 18 |
| S24 | Cervical dilatation.mp. | 412 |
| S25 | Labor progress* | 1,435 |
| S26 | Head descent | 37 |
| S27 | S23 or S24 or S25 or S26 | 2,136 |
| S28 | S5 and S22 and S27 | 10 |

OVID-Medline

| # | Query |  |
| --- | --- | --- |
| S1 | Labor and women.mp. | 31,231 |
| S2 | Delivery and women.mp. | 71,570 |
| S3 | Childbirth and women.mp. | 11,922 |
| S4 | Intrapartum and women.mp. | 3,806 |
| S5 | S1 or S2 or S3 or S4 | 103,231 |
| S6 | Non-invasive assessment.mp. | 2120 |
| S7 | Routine vaginal exam.mp. | 14 |
| S8 | Purple line.mp. | 13 |
| S9 | Behavior observation.mp. | 616 |
| S10 | Uterine contraction.mp. | 8,319 |
| S11 | Verbal expression.mp. | 329 |
| S12 | Electrohysterography | 85 |
| S13 | Electrohysterogram | 104 |
| S14 | Uterine electromyography | 52 |
| S15 | Uterine monitoring | 333 |
| S16 | Uterine contraction | 8,319 |
| S17 | External tocodynamometer | 19 |
| S18 | Transperineal ultrasond | 551 |
| S19 | Transperineal sonography | 80 |
| S20 | Transabdominal ultrasond | 1,068 |
| S21 | Transabdominal sonography | 295 |
| S22 | S6 or S7 or S8 or S9 or S10 or S11 or S12 or S13 or S14 or 15 or S16 or S17 or S18 or S19 or S20 or S21 | 13,574 |
| S23 | Fetal descent.mp. | 42 |
| S24 | Cervical dilatation.mp. | 1,863 |
| S25 | Labor progress* | 177 |
| S26 | Head descent | 50 |
| S27 | S23 or S24 or S25 or S26 | 2,073 |
| S28 | S5 and S22 and S27 | 157 |
