# Supplementary material for: Accuracy of non-invasive methods for assessing the progress of labor in the first stage: a systematic review and meta-analysis
Source: BMC Pregnancy Childbirth. 2022 Aug 1;22:608. doi: 10.1186/s12884-022-04938-y (PMC9341104; doi:10.1186/s12884-022-04938-y)
Supplement: Supplementary file 2 — Additional file 2. Study Quality of Evidence According to Guidelines. [file 12884_2022_4938_MOESM2_ESM.docx]

**Additional file 2** Study Quality of Evidence According to Guidelines

| Certainty assessment | No. of Studies (No. of Patients) | Study Design | Domain | | | | | |
| --- | --- | --- | --- | --- | --- | --- | --- | --- |
|  |  |  | Risk of Bias | Indirectness | Inconsistency | Imprecision | Publication  Bias | Test of Accuracy |
|  | | | |  |  |  |  |  |
| Appearance assessment | 2 (470) | cross-sectional (cohort type accuracy study) | very serious ^a, b^ | not serious | serious ^c^ | serious ^e^ | Undetected ^f^ | ⨁◯◯◯ VERY LOW |
| Occiput-spine angle | 2 (730) | cross-sectional (cohort type accuracy study) | serious ^a^ | not serious | serious ^c^ | serious ^e^ | Undetected ^f^ | ⨁◯◯◯ VERY LOW |
| Head-perineum distance | 8 (1330) | cross-sectional (cohort type accuracy study) | serious ^a^ | not serious | serious ^d^ | not serious | Undetected ^f^ | ⨁⨁◯◯ LOW |
| Angle of progression | 9 (1394) | cross-sectional (cohort type accuracy study) | serious ^a^ | not serious | serious ^d^ | not serious | Undetected ^f^ | ⨁⨁◯◯ LOW |
| Fetal head direction | 1(70) | cross-sectional (cohort type accuracy study) | serious ^a^ | not serious | serious ^c^ | not serious | Undetected ^f^ | ⨁⨁◯◯ LOW |
| Head Symphysis Distance | 1(201) | cross-sectional (cohort type accuracy study) | serious ^a^ | not serious | serious ^c^ | not serious | Undetected ^f^ | ⨁⨁◯◯ LOW |

GRADE = Grades of Recommendation Assessment, Development, and Evaluation

a. Convenience sample with an unknown proportion of representativeness of the population of subjects enrolled.

b. No blinding to the index test in one study.

c. Only one or two studies are available; indicative that the literature is not well established.

d. Unexplained heterogeneity between studies.

e. High false- negatives in one study.

f. Due to zero events in both study groups, a relative risk could not be estimated.
