# Supplementary material for: Accuracy of non-invasive methods for assessing the progress of labor in the first stage: a systematic review and meta-analysis
Source: BMC Pregnancy Childbirth. 2022 Aug 1;22:608. doi: 10.1186/s12884-022-04938-y (PMC9341104; doi:10.1186/s12884-022-04938-y)
Supplement: Supplementary file 3 — Additional file 3. Sensitivity and specificity row data. [file 12884_2022_4938_MOESM3_ESM.docx]

**Additional file 3**

1. **Raw data of purple line**

| Author | Year | Assessment | Accuracy  (%) | Sensitivity  (%) | Specificity  (%) | PPV  (%) | True positives | True negatives | False positives | False negatives |
| --- | --- | --- | --- | --- | --- | --- | --- | --- | --- | --- |
| Eid Farrag and Abd ElHamed Eltohamy | 2021 | purple line | 85 | 88 | 40 | 85 | 84 | 10 | 15 | 11 |
| Kordi | 2013 | purple line | 81 | 69 | 43 | 85 | 199 | 26 | 35 | 89 |
|  |  | transvers diagonal of Michaelis Sacral | 80 | 89 | 59 | 85 | 255 | 57 | 40 | 28 |

Note: PPV, positive predictive value;

1. **Raw data of occiput-spine angle**

| Author | Year | Cut-off points | Sensitivity  (%) | Specificity  (%) | PPV  (%) | True positives | True negatives | False positives | False negatives |
| --- | --- | --- | --- | --- | --- | --- | --- | --- | --- |
| Mukdee | 2021 | ≥ 100° | 84 | 17 | 88 | 244 | 7 | 46 | 33 |
| Maged | 2019 | ≥ 126° | 82 | 65 | 45 | 85 | 193 | 19 | 104 |

Note: PPV, positive predictive value

1. **Raw data of head-perineum distance**

| Author | Year | Assessment | Cut-off  Points (mm) | Sensitivity  (%) | Specificity  (%) | PPV  (%) | True positives | True negatives | False positives | False negatives |
| --- | --- | --- | --- | --- | --- | --- | --- | --- | --- | --- |
| Hjartardóttir | 2021 | 3D | 46 | 67 | 67 | 87 | 51 | 15 | 8 | 25 |
| Ibrahim | 2021 | 2D | 39.5 | 62 | 63 | 85 | 287 | 86 | 51 | 176 |
| Fahmy | 2020 | 2D | 40 | 96 | 84 | 92 | 39 | 18 | 3 | 2 |
| Kandil | 2020 | 2D | 45 | 88 | 91 | 96 | 50 | 21 | 2 | 7 |
| Solaiman | 2020 | 2D | 42 | 80 | 84 | 85 | 12 | 11 | 2 | 3 |
| Wiafe | 2018 | 2D | 36 | 79 | 72 | 49 | 42 | 111 | 42 | 10 |
| Eggebø | 2014 | 2D &3D | 40 | 69 | 82 | 92 | 78 | 31 | 7 | 35 |
| Torkildsen(2D) | 2011 | 3D | 40 | 62 | 85 | 93 | 52 | 22 | 4 | 32 |
| Torkildsen(3D) | 2011 | 2D | 40 | 61 | 81 | 91 | 51 | 21 | 5 | 33 |

Note: 2D, two-dimensional ultrasound; 3D, three-dimensional ultrasound; PPV, positive predictive value

1. **Raw data of Angle of progression**

| Author | Year | Assessment | Cut-off points | Sensitivity  (%) | Specificity  (%) | PPV  (%) | True positives | True negatives | False positives | False negatives |
| --- | --- | --- | --- | --- | --- | --- | --- | --- | --- | --- |
| Elkadi | 2021 | 2D | 97° | 92 | 80 | 98 | 47 | 4 | 1 | 4 |
| Hjartardóttir | 2021 | 3D | 93° | 79 | 46 | 82 | 59 | 11 | 13 | 16 |
| Ibrahim | 2021 | 2D | 106.5° | 52 | 81 | 90 | 239 | 113 | 27 | 221 |
| Fahmy | 2020 | 2D | 120° | 92 | 84 | 92 | 43 | 20 | 4 | 4 |
| Kandil | 2020 | 2D | 104° | 90 | 86 | 95 | 54 | 17 | 3 | 6 |
| Solaiman | 2020 | 2D | 115° | 93 | 84 | 87 | 14 | 11 | 2 | 1 |
| Wiafe | 2018 | 2D | 101° | 68 | 68 | 40 | 32 | 105 | 49 | 15 |
| Eggebø | 2014 | 2D &3D | 110° | 68 | 72 | 88 | 77 | 27 | 10 | 36 |
| Torkildsen(2D) | 2011 | 3D | 110° | 56 | 75 | 87 | 46 | 21 | 7 | 36 |
| Torkildsen(3D) | 2011 | 2D | 110° | 65 | 75 | 88 | 53 | 22 | 7 | 28 |

Note: 2D, two-dimensional ultrasound; 3D, three-dimensional ultrasound; PPV, positive predictive value

1. **Diagnostic accuracy of HD, HSD**

| Author | Year | Assessment | Cut-off points | Sensitivity  (%) | Specificity  (%) | PPV  (%) | True positives | True negatives | False positives | False negatives |
| --- | --- | --- | --- | --- | --- | --- | --- | --- | --- | --- |
| Fahmy | 2020 | HD | 30° | 96 | 92 | 96 | 45 | 21 | 2 | 2 |
| Wiafe | 2018 | HSD | 28 mm | 75 | 71 | 44 | 35 | 109 | 45 | 12 |

Note: fetal head direction, HD; head symphysis distance, HSD; NPV, negative predictive value; PPV, positive predictive value
